# Low-frequency ultrasound for pulmonary hypertension therapy

**DOI:** 10.1186/s12931-024-02713-5

**Published:** 2024-02-05

**Authors:** Vytautas Ostasevicius, Vytautas Jurenas, Mantas Venslauskas, Laura Kizauskiene, Vilma Zigmantaite, Edgaras Stankevicius, Algimantas Bubulis, Joris Vezys, Sandra Mikuckyte

**Affiliations:** 1 Institute of Mechatronics, Kaunas University of Technology, Studentu Street 56, 51424 Kaunas, Lithuania; 2 Department of Computer Sciences, Kaunas University of Technology, Studentu Street 50, 51368 Kaunas, Lithuania; 3 Biological Research Center Lithuanian, University of Health Sciences, Tilžės Street 18, 47181 Kaunas, Lithuania; 4 Laboratory of Membrane Biophysics, Cardiology Department, Lithuanian University of Health Sciences, Sukilėlių Street 15, 50103 Kaunas, Lithuania; 5 Institute of Physiology and Pharmacology, Lithuanian University of Health Sciences, A. Mickevicius Street 9, 44307 Kaunas, Lithuania; 6 Department of Mechanical Engineering, Kaunas University of Technology, Kaunas University of Technology, Studentu Street 56, 51424 Kaunas, Lithuania

## Abstract

**Background:**

Currently, there are no reliable clinical tools that allow non-invasive therapeutic support for patients with pulmonary arterial hypertension. This study aims to propose a low-frequency ultrasound device for pulmonary hypertension therapy and to demonstrate its potential.

**Methods:**

A novel low-frequency ultrasound transducer has been developed. Due to its structural properties, it is excited by higher vibrational modes, which generate a signal capable of deeply penetrating biological tissues. A methodology for the artificial induction of pulmonary hypertension in sheep and for the assessment of lung physiological parameters such as blood oxygen concentration, pulse rate, and pulmonary blood pressure has been proposed.

**Results:**

The results showed that exposure of the lungs to low-frequency ultrasound changed physiological parameters such as blood oxygen concentration, pulse rate and blood pressure. These parameters are most closely related to indicators of pulmonary hypertension (PH). The ultrasound exposure increased blood oxygen concentration over a 7-min period, while pulse rate and pulmonary blood pressure decreased over the same period. In anaesthetised sheep exposed to low-frequency ultrasound, a 10% increase in SpO_2_, a 10% decrease in pulse rate and an approximate 13% decrease in blood pressure were observed within 7 min.

**Conclusions:**

The research findings demonstrate the therapeutic efficiency of low-frequency ultrasound on hypertensive lungs, while also revealing insights into the physiological aspects of gas exchange within the pulmonary system.

## Introduction

Pulmonary hypertension (PH) is a disease which is almost incurable and has a high mortality rate. PH is characterized by an increase in blood pressure > 20 mm Hg in the right ventricle of the heart and therefore requires medical attention for such patients [1]. Traditional drugs used to treat pulmonary hypertension include prostacyclin analogues and receptor agonists, phosphodiesterase 5 inhibitors, endothelin receptor antagonists and cGMP activators. None of these drugs cure pulmonary hypertension, and median survival remains below 3 years after diagnosis. Significant research efforts have led to the emergence of innovative therapies such as stem cell therapy, gene transfer and epigenetic therapy [2–4]. The research results reported in [2] state that stem cells successfully eliminate pulmonary vascular endothelial dysfunction, excessive proliferation of pulmonary arterial smooth muscle cells, and mitochondrial dysfunction in patients with pulmonary hypertension. The study in [3] provides an overview of the currently used in vitro gene delivery methods for pulmonary vascular cells and describes some recent advances in gene delivery for pulmonary hypertension in vivo. Epigenetics refers to heritable changes in chromatin that lead to changes in gene expression without changing the DNA sequence. Restoration or reduction of gene expression by therapeutic delivery of target genes has been shown to treat and prevent pulmonary hypertension or inhibit disease progression [4]. The described innovative methods of pulmonary hypertension therapy are invasive and expensive, requiring complex techniques and qualifications. Our goal is to demonstrate the possibilities of low-frequency ultrasound in solving this problem. Diagnostic ultrasound is used to assess health conditions, while therapeutic ultrasound is used to treat health problems. The safety of ultrasound exposure is crucial to the patient's well-being. According to the standard of World Federation for Ultrasound in Medicine and Biology, and by the Food and Drug Administration (USA), the acoustic intensity emitted by medical ultrasound diagnostic equipment must not exceed 720 mW/cm^2^ in the frequency range of 1–10 MHz [5]. Experiments on the effect of ultrasound on fat lipolysis have shown that at acoustic intensities of 800–1000 mW/cm^2^ and an ultrasound frequency of 1 MHz, the effective depth, or live body fat lipolysis, is 0.9 cm for muscle and 1.7 cm for fat [6], while for biological tissue, the penetration depth of ultrasound at this frequency is 3–5 cm, which increases with decreasing ultrasound frequency. The acoustic intensity of ultrasound decreases exponentially as it penetrates deeper into biological tissue. The biological tissue attenuation coefficient is proportional to the frequency of ultrasound applied to the human body. It means that low-frequency (20–100 kHz) ultrasound would be suitable to therapeutically affect deeper internal organs. Although a non-invasive ultrasound study carried out in the article [7] was conducted to estimate the influence of acoustic signal using a pair of transducers or a belt around the human chest with 12 sensors spaced 5 cm apart and one pulse transmitter attached to the sternum, such a set of the ultrasound transducers could also be applied to therapy, which is another field of application of ultrasound [8–13]. Ultrasound can cause several non-thermal and thermal effects on biological tissues [8]. The article [9] focuses on vascular effects and microbubble-induced exposure for drug delivery, with an assessment of increased vascular injury and occlusion. Another paper [10] describes a study in which seventeen patients and nine healthy individuals were assessed using body plethysmography and low-frequency ultrasound. Clinical ultrasound experiments on animals have been conducted for many years [11]. Animal models are required to determine the metabolism in PH [12]. In this article dependence of sound propagation through damaged lungs of pigs on expiratory pressure was evaluated.

The device for low frequency ultrasound therapy [13] is a traditional Langevin-type transducer operating in first longitudinal vibration mode. Therefore, a low-frequency (less than 100 kHz) ultrasound wave transducer, operating in a higher vibration mode, has been developed [14], and the results of hypertensive sheep in vivo studies are presented in this paper. In contrast to commonly used transducers for sonication, that exclusively generate only longitudinal vibration modes, our newly developed transducer operates at radial vibration mode. This shift has led to enhanced acoustic signal penetration, made the signal less scattered and allowed us to increase the acoustic effect on deeper biological tissues, consequently adapting the developed device for therapeutic applications.

## Materials and methods

### Design of the proposed low frequency ultrasonic acoustic wave transducer

**Fig. 1:**
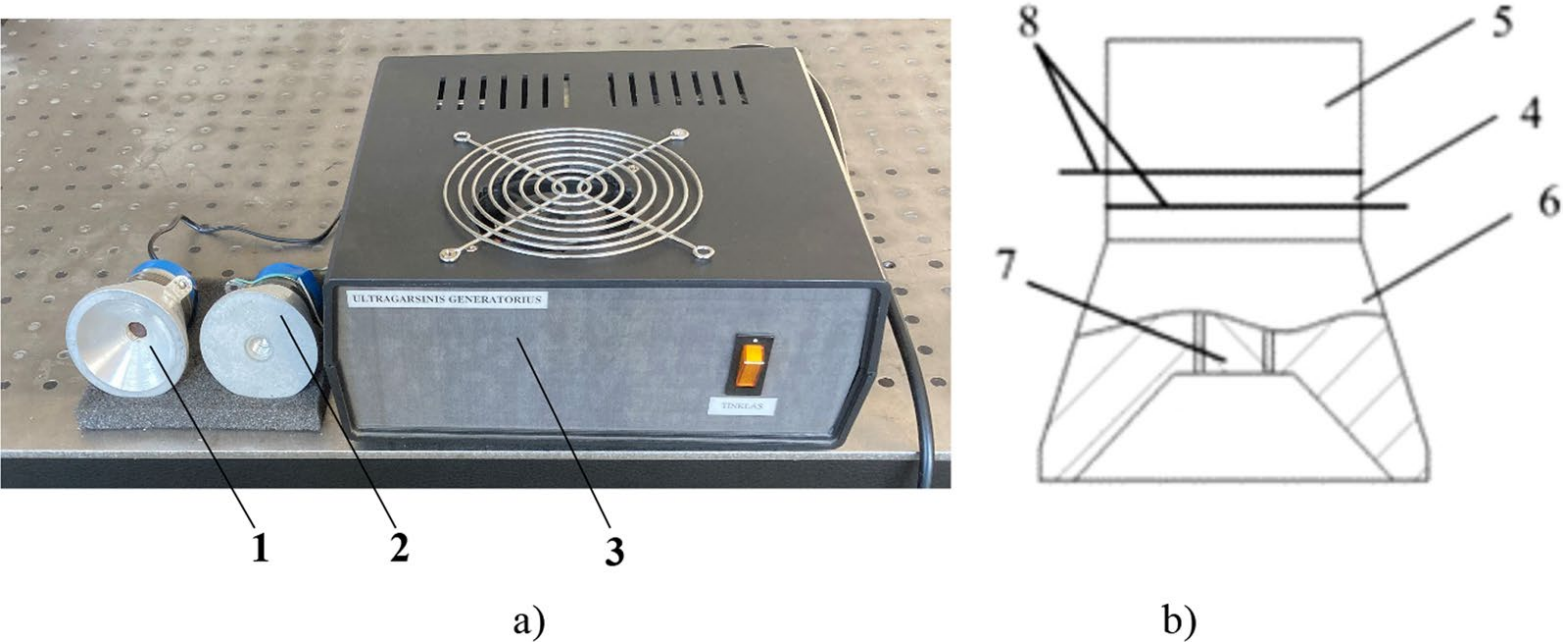
**a** Ultrasonic transducer with a cut-out (1) and flat (2) surfaces with laboratory-made controller (3); **b** layout of the ultrasonic transducer with a cut-out surface: 4—ring-shaped piezoelectric element, 5—back mass, 6—front mass, 7—a preload bolt, 8—copper-nickel electrodes

Langevin-type [15] piezoelectric transducers with cut-out and flat surfaces of the front mass have been developed to evaluate the therapeutic effects of low frequency sonophoresis on biological tissues (Fig. 1).

**Fig. 2:**
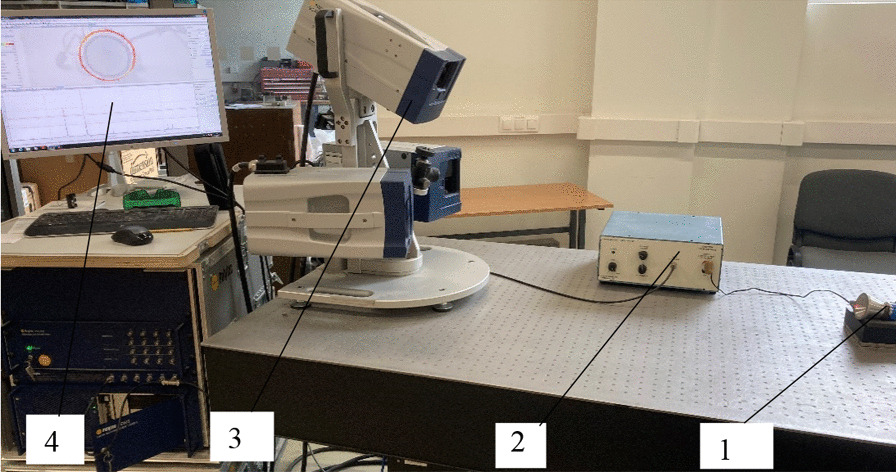
Setup for modal analysis: 1—transducer; 2—amplifier EPA-104 (PiezoSystem Inc., USA); 3—Polytec scanning laser head; 4—Polytec data acquisition system/signal generator

The transducer consists of two piezoelectric ring-shaped ceramic parts 4 sandwiched between two metallic masses, called the back mass 5 and the front mass 6. These masses are held in place by a preload bolt 7 which allows the ceramics to be preloaded in order to avoid undesirable tensile stresses. The back mass and the preload bolt were made of durable high impedance steel SUS 304. The front mass was made of aluminium since it has a lower acoustic impedance than piezoelectric elements and has good acoustic radiation characteristics. Two ring-shaped PZT-4 type piezoelectric ceramic elements 4 were sandwiched between two solid masses 5 and 6, and two 0.5 mm thick copper-nickel electrodes 8 provided the input to the piezoelectric ceramic cells. The ultrasound was transmitted through the sheep's body using two using two different transducers. One transducer had a cut-out front mass surface (Fig. 1, a, 1), and the other had a standard flat front mass surface (Fig. 1, a, 2).

### Methods

The study with sheep pulmonary arteries was conducted according to the principles defined in the Declaration of Helsinki [16]. Permission to perform experimental research with animals was obtained from the Lithuanian experimental animal ethics board by Lithuanian veterinary and food services (Issued date 2022-02-02; Nr. G2-195).

The sheep were first sedated using Xylazine hydrochloride (1 mg/kg intramuscular (IM), Bela–Pharm GmbH & Co.KG, Germany) as premedication, then transferred to the operating table and injected with Butorphanol (0.2 mg/kg IM, Richter Pharma, Austria). The sheep were then shaved, hair was removed from both sides of the chest, sternum, jugular vein, and left muzzle. Under sterile conditions, venous catheters (16 G, Provein™, Lars Medicare, India) were inserted into the left jugular vein. Saline solution was injected intravenously at a rate of 5 ml/kg/h. Ketamine (7 mg/kg intravenous (IV)) was used to induce anaesthesia and sheep were prepared for intubation. The larynx then was visualised through a laryngoscope and was sprayed with 10% lidocaine spray solution (Egis Pharmaceuticals PLC, Hungary) to avoid respiratory spasm, endotracheal tube (ET, Kruuse, Denmark) was secured, the cuff inflated, and artificial lung ventilation performed using ventilator (model Fabius Tiro, Draeger, Germany). Long-term deep anaesthesia was achieved using inhalation gas Sevoflurane 2% (Baxter, Belgium) and maintained with Midazolam (0.1 mg/kg/h, Kalceks, Latvia) and fentanyl (0.05 mg/kg/h, Kalceks, Latvia) using continuous rate infusion. The oxygen level was adjusted by about 21% to keep the animal adequately oxygenated during anaesthesia. The sheep were carefully monitored using both manual practices and mechanical tools: eye position, palpebral reflex, mucous membrane colour and capillary refill time were checked and recorded at 5-min intervals.

## Results

### Comparison of the dynamics of conventional and engineered low-frequency ultrasonic acoustic wave transducers

A Polytec laser Doppler 3D-scanning vibrometer PSV-500-3D-HV (Polytec GmbH, Germany) was used for the modal analysis of the front surfaces of the cut-out and flat transducers (Fig. 2).

**Fig. 3:**
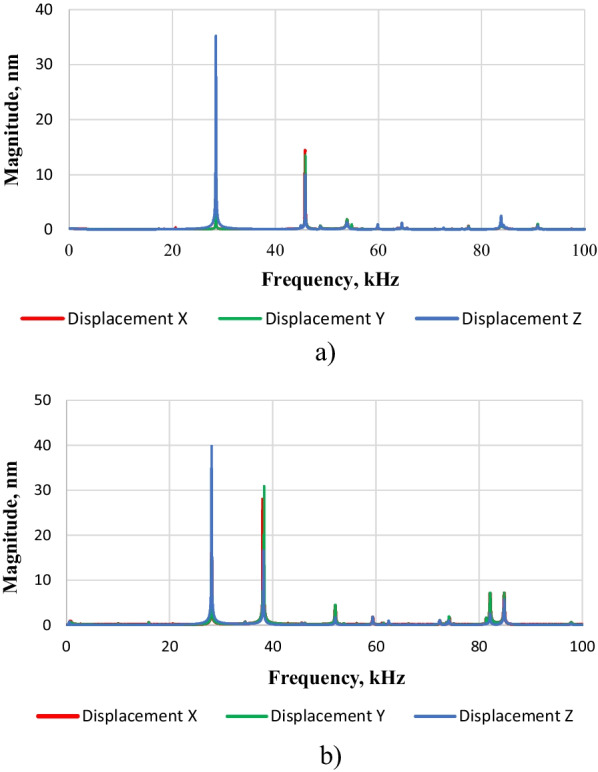
Frequency responses of the two developed transducers: **a** with flat and **b** with cut-out surfaces; blue—out-of-plain vibration, red and green—in-plain vibrations

When the transducers were excited with 10 V_*P-P*_ sinusoidal (10–100) kHz frequency signals, their natural oscillation modes were recorded (Fig. 3a, b). The transducer with a flat surface has a longitudinal mode with an amplitude of 35.6 nm at 28.44 kHz and a radial mode with an amplitude of 14.2 nm at 45.81 kHz. The transducer with a cut-out surface has a longitudinal mode with an amplitude of 39.9 nm at 28.13 kHz and a radial mode with an amplitude of 30.1 nm at 38.19 kHz. The measured results show that higher vibration amplitudes are generated by a transducer with a cut-out annular surface of the front mass.

**Fig. 4:**
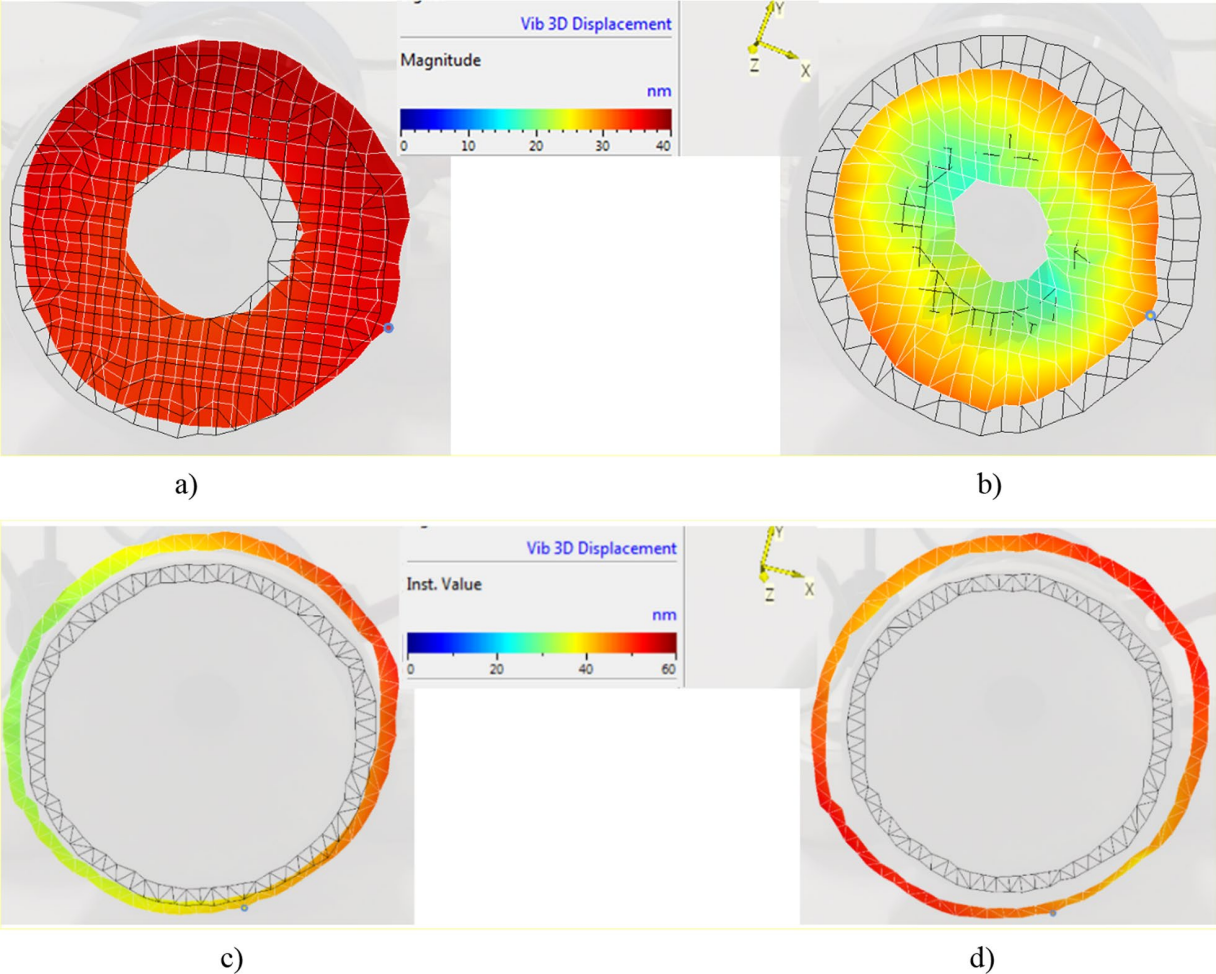
The eigenmodes at the resonant frequencies: **a** for transducer with flat surface at frequencies of 28,44 kHz; **b** transducer with flat surface at frequency of 45,81 kHz; **c** transducer with cut-out surface at frequency of 28,13 kHz; **d** transducer with cut-out surface at frequency of 38,19 kHz

Three-dimensional scanning vibrometer was used to determine the operating deflection shapes and eigenmodes of the developed ultrasound transducer in the frequency range from 0 to 100 kHz. The vibrometer was used to measure the deflection shapes of the transducer’s front mass surface at the resonant frequencies of the longitudinal and radial vibration modes and is shown in Fig. 4.

The eigenmodes (Fig. 4) show that the flat transducer has its largest displacements at 28 kHz and 46 kHz, while the cut-out transducer—at 28 kHz and 38 kHz, respectively. Hence, the efficiency of ultrasonic oscillations for micro/macro circulatory structures can be increased by using piezoelectric acoustic transducers with a cut-out surface that can be excited by a higher frequency mode. This cut-out surface transducer has been found to have a more directional overall sound pressure compared to a flat surface, which is very important in real-world applications. The effectiveness of PH therapy is attributed to its ability to stimulate a directed and deeply penetrating acoustic wave that precisely affects the part of the body to be treated.

### Measurement of the ultrasound pressure

In the experimental studies using ultrasound, a hydrophone was used to measure the acoustic wave pressure and to determine its intensity. Knowing the pressure, velocity, and density of the medium in which this acoustic wave propagates, we can calculate the intensity of the acoustic wave.

**Fig. 5:**
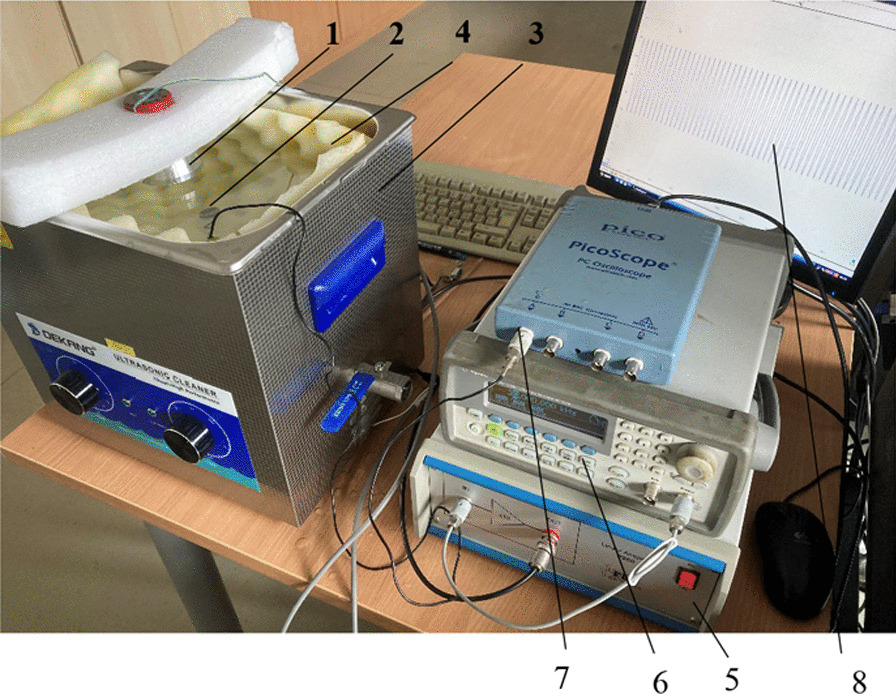
A set up for measurement of the acoustic wave pressure generated by the ultrasonic transducer: 1—ultrasonic transducer; 2—hydrophone; 3—water bath; 4—ultrasound attenuating material; 5—power amplifier P200 (FLC Electronics AB, Sweden); 6—signal generator Agilent 33220A; 7—oscilloscope PicoScope 3424 (PicoTechnology Ltd., UK) 8—PC

Before studying ultrasound penetration into biological tissues, it is important to know the intensity of the ultrasonic signal emitted by the transducer. For this purpose, ultrasound level measurement equipment has been developed (Fig. 5). This figure presents the setup for the measuring the acoustical pressures emitted by transducer 1 immersed in a water bath 3, oscillating at the second vibrational mode of 38 kHz. In order to suppress the reflected ultrasonic waves from the walls of the bath, an ultrasound-attenuating material 4 was used as a lining. Two different hydrophones were used in the experiment. Calibration of a broad-spectrum piezoelectric hydrophone 2 with an active diameter of 15 mm was performed using a hydrophone HCT-0320, coupled with a digital pressure meter MCT-2000 (Onda Corp., USA) in the frequency range of 20–100 kHz. The tests were carried out with transducers excited by a harmonic electrical signal of 50 V_*P-P*_.

The waves generated by an acoustic transducer can be used to create a sound field, which can be divided into distinct areas called near and far fields. Consequently, within the near field the interference pattern engenders spatial variations in sound pressure levels. As the waves propagate away from the transducer, the sound field gradually equalizes, leading to enhanced predictability and amplification of the emitted waves in the region beyond the near field, often referred to as the "natural focus" [17].

The near-field distance in water of the investigated transducer with a diameter of 40 mm and an operational frequency of 38 kHz is greater than 10 mm. Therefore, the distance between the transducer and the hydrophone was set to more than 10 mm, for the measurements in water and the measured acoustic pressure was determined as the peak output value emitted by the investigated transducer. Characterization of the acoustic fields generated by transducers is necessary for the development of standards that ensure a positive risk–benefit outcome of treatment.

The expression for acoustic intensity given in IEC 61102 assumes that the measurements were made in the far field of the transducer. Therefore, when measurements were performed in water, the distance between the transducer and the hydrophone was set to more than 10 mm, and the measured acoustic pressure and intensity values were determined as peak input values transmitted through the sonicated tissue [17].

The ability of an ultrasound wave to transfer from one type of tissue to another depends on the difference in impedance between two different biological tissues and ultrasound attenuation factor. If the impedance is similar, a large part of the incident sound intensity will be transmitted through the boundary interface; if the difference is large, the sound is reflected. As indicated in Table 1, bone, soft tissue, and air correspond to the typical ranges of high, medium, and low biological tissue acoustic impedance values. Approximately 1% of the ultrasound intensity is reflected at the fat-muscle interface, with nearly 99% of the intensity being transmitted to deeper biological tissues. Almost 100% of the intensity is reflected at the air-muscle interface. A Transonic G-15 gel placed between the sheep's skin and the transducer is an important part of the standard ultrasound procedure to ensure a good transducer-skin interface by eliminating air that would reflect the ultrasound.

**Table 1:** Acoustic parameters of ultrasound in various materials and biological tissues

Tissue	Density, kg/m^3^	Longitudinal wave velocity, m/s	Acoustic impedance (kg/m^2^ s) × 10^6^	Attenuation coefficient dB/cm at 1 MHz
Air	1.2	330	0.0004	7.50
Water	1000	1480	1.48	0.0022
Blood	1060	1560	1.62	0.15
Skin	1150	1730	1.99	0.35
Liver	1060	1550	1.64	0.50
Heart	1040	1560	1.62	0.52
Skeletal muscles:	1070	1590	1.70	
Parallel fibres				1.40
Perpendicular fibres				0.96
Bone	1380–1810	2700–4100	3.75–7.38	15.0
Lung	400	440–500	0.18–0.20	40.0

The attenuation coefficient depends on the density and viscosity of the material and the frequency of the ultrasound. The acoustic properties of soft biological tissues, bones and gas inclusions depend on the attenuation coefficient of the acoustic wave passing through these tissues. The attenuation coefficients measured in various biological tissues are reported in the literature [18, 19] (Table 1).

**Fig. 6:**
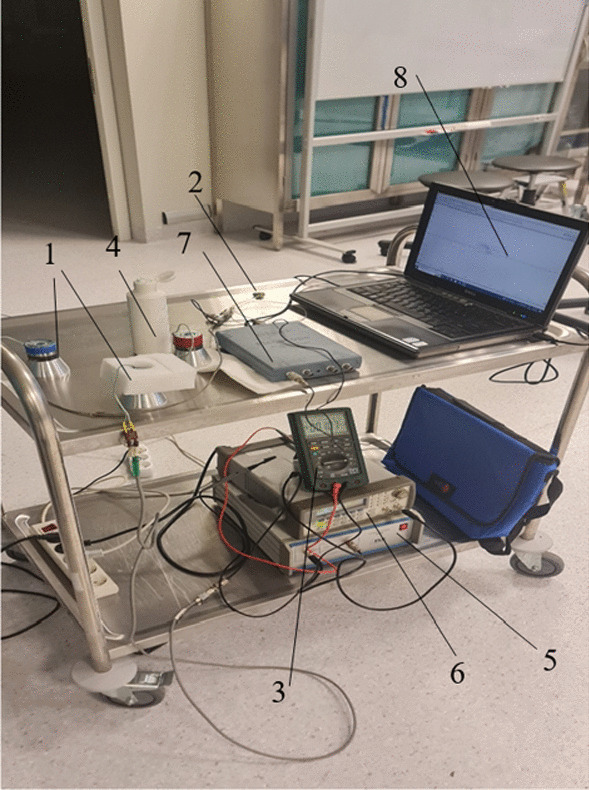
Experimental setup for measurement of the sonication intensity transmitted through tissue by an ultrasonic transducer: 1—ultrasonic transducer; 2—hydrophone; 3—RMS multimeter MS8218 (Mastech Group Ltd.); 4—ultrasonic gel Transonic G-15 (TELIC, S.A.U., Spain); 5—power amplifier P200 (FLC Electronics AB, Sweden); 6—signal generator Agilent 33220A; 7—oscilloscope PicoScope 3424 (PicoTechnology Ltd., UK); 8—PC

**Fig. 7:**
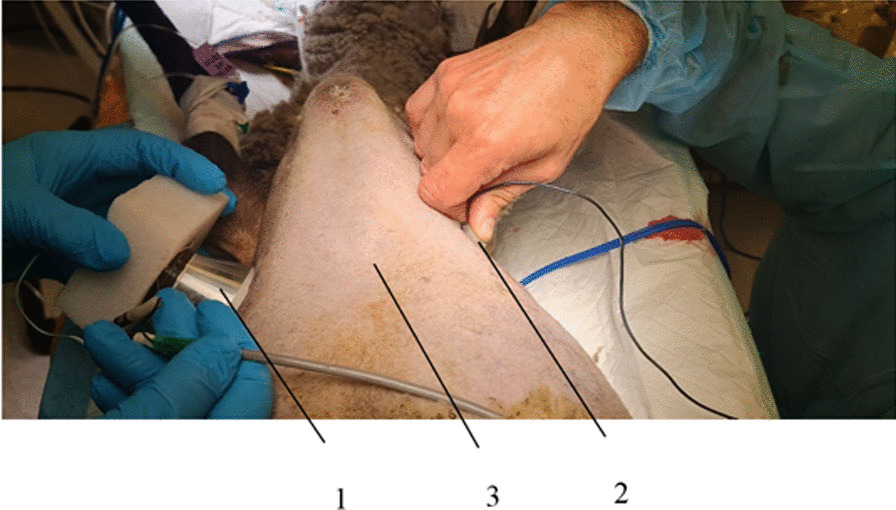
Measurement of the transmitted ultrasound signal through the sheep’s body: 1—transducer; 2—hydrophone; 3—a sheep’s body

The stand shown in Fig. 6 was set up for an experimental study of in vivo ultrasound transmission through the body of a sheep. The sinusoidal signal was excited by the generator 6 with an amplitude of 5 V_*P-P*_ at the resonance frequency of the investigated transducers and was augmented by a power amplifier 5 with a constant voltage gain of 10. The ultrasound transducer was positioned on the sheep’s chest near the lungs at right angles to the ribs and sent ultrasonic waves across a sheep body, which were received by a piezoelectric hydrophone and transmitted to a computer screen through an oscilloscope Pico Scope 3424 (see Figs. 6 and 7).

### Pulmonary hypertension study in sheep

Studies of ultrasound signal transmission through the body of the sheep and its effect on pulmonary hypertension were performed in live sheep (Fig. 7). Noradrenaline, or norepinephrine, is important for the body's therapeutic response. As a drug, it produces effects such as increased blood pressure and pulse rate, widening of the pulmonary airways, and constriction of blood vessels in nonessential organs. The ultrasound was transmitted through the sheep's body, and the acoustic pressure was measured with two different transducers. One transducer had a cut-out front mass surface (Transducer 1), and the other had a standard flat front mass surface (Transducer 2). The test was carried out by exciting the transducers with an electrical signal of 50 V_*P-P*,_ and the input electrical power used by the transducers was about 20 W. The transducer with a cut-out surface was excited at a frequency of 38 kHz and the transducer with flat surface was excited at 46 kHz. The acoustic pressure transmitted through the body of a sheep was measured with a hydrophone, and the results are presented in Table 2.

**Table 2:** An approximate acoustic pressure transmitted by the transducer

Transducer type	Acoustic pressure in water kPa	Acoustic pressure through sheep’s body kPa	Acoustic intensity in water mW/cm^2^	Acoustic intensity transmitted through sheep’s body mW/cm^2^
Transducer 1	75	1.5	380.07	0.24
Transducer 2	54	0.9	197.03	0.06

From this table, it is evident that the technical parameters of the modified transducer with a cut-out surface, such as acoustic pressure through sheep's body is ~ 1.5 times, acoustic intensity in water ~ 2.0 times, and acoustic intensity transmitted through sheep's body ~ 4.0 times higher than the parameters of the transducer with a standard flat surface.

The frequencies of the second mode oscillations of the investigated transducers (transducer with a standard flat surface – 46 kHz, transducer with a cut-out surface – 38 kHz) differ little and the characteristics of the acoustic fields they create can be compared, regardless of the small difference in frequencies. The research results show that the output surface of the modified transducer generates a larger vibration amplitude and due to its circular shape, the acoustic field spreads more directionally.

The permeability of standard flat and cut-out front mass surface actuators through the sheep's body were compared.

To induce pulmonary hypertension and to measure blood pressure changes in the right ventricle of the sheep's heart under ultrasound, it was necessary to open the thorax after the sheep had been put under general anaesthesia. The chest was opened for the sheep, because it was necessary to insert a catheter directly into the pulmonary artery to accurately measure blood pressure changes in the right ventricle of the sheep's heart. During tests with 5 sheep, pulmonary hypertension was artificially induced applying vessel ligation and changes in blood oxygen concentration were measured under the influence of low-frequency ultrasound. Measurements of sheep pulse rate, pulse oximetry, capnography, IBP and NIBP were performed with a multifunctional monitoring device (Draeger Vista 120, Draeger, Germany). IBP was measured in the left facial artery.

**Fig. 8:**
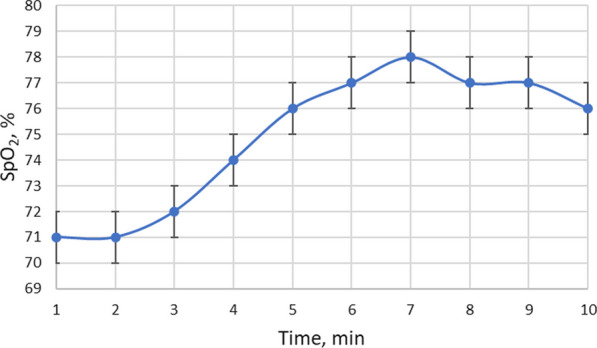
Variation in oxygen concentration in the blood of sheep under the influence of low-frequency ultrasound

The duration of the process was determined empirically in real-time by determining the effect of ultrasound on the measured physiological parameters of the tested sheep. The testing process lasted for 10 min, and the sonication was stopped after 7 min. The ultrasound effect was determined and then the ultrasound transducer was removed. The dispersion intervals of the values of the measured physiological parameters of the five tested sheep are shown graphically for the individual measurement points. The changes in the oxygen concentration in the lungs of the sheep (Fig. 8) show that an increase of the parameter SpO_2_ begins after 2 min of the sonication. After 7 min of exposure to ultrasound, the oxygen concentration in the blood increases by more than 10%. When the ultrasound is stopped, a slight decrease in the oxygen concentration is observed for the next 3 min, until the end of the test.

**Fig. 9:**
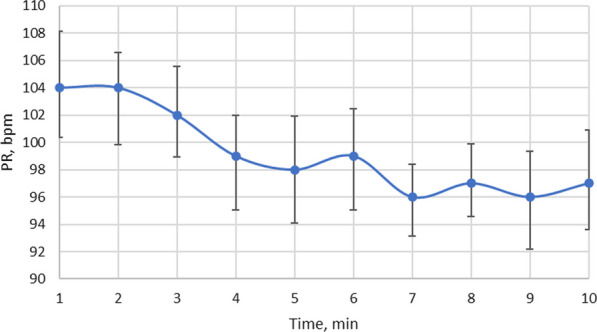
Variation in sheep pulse rate under low-frequency ultrasound

During the tests, the sheep's pulse rate was measured (Fig. 9). The time on the x-axis is the time after start of sonication. It is evident from this graph that within 7 min of exposure to low-frequency ultrasound, the pulse rate is decreased by approximately 10 percent. After stopping the ultrasound radiation for the next 3 min., slight fluctuations in sheep pulse rate were observed until the end of the tests.

**Fig. 10:**
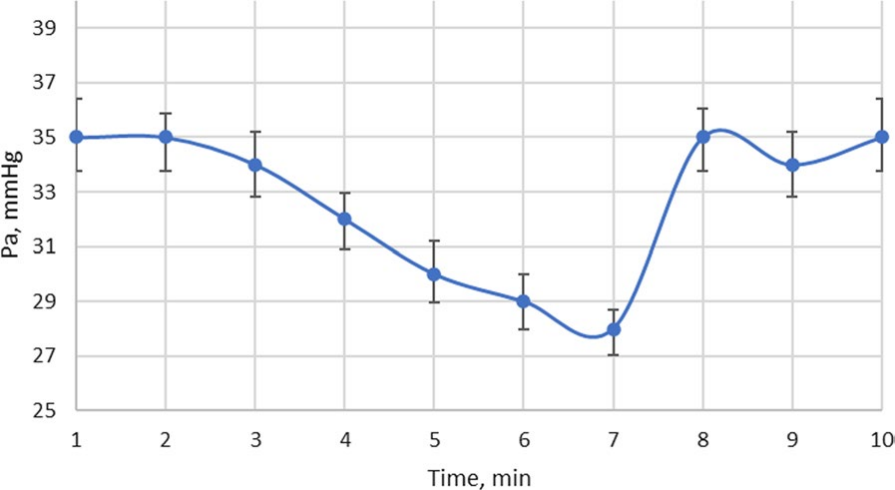
Changes in blood pressure in sheep lungs under low-frequency ultrasound

Blood pressure in the lungs was also measured (Fig. 10). It is evident from graph (Fig. 10) that within 7 min of exposure to low-frequency ultrasound, there is a reduction in blood pressure of approximately 13%. After stopping the ultrasound for the next 3 min and staying in place with the transducer and hydrophone as shown in Fig. 7 until the end of the tests, recovery of blood pressure to the pre-test value was observed.

## Discussion

Despite modern medical achievements and advanced treatment options, pulmonary arterial hypertension remains an intractable disease with a very high mortality rate. This disease is usually asymptomatic and only becomes apparent when most of the distal pulmonary arteries have become obliterated. Obliteration occurs due to reduced pulmonary arterial compliance and increased deposition of extracellular matrix/collagen in the pulmonary arteries in the presence of hypertension. To date, the quality of life of patients has been improved by prescribing diuretics, anticoagulants, vasodilators, etc. However, dosing, and other problems often arise with these drugs, which are frequently addressed by changing the treatment paradigm for pulmonary hypertension from pharmacological to non-invasive approaches, among which a low-frequency ultrasound transducer for the treatment of artificially induced PH in sheep has been proposed in this study. When it comes to humans, PH manifests as chronic pain. Therefore, our proposed low-cost method that does not use drugs that may have side effects on internal organs should reduce pain in this population [20]. As the main function of the lungs is to supply oxygen to the blood, maintaining the level of oxygen concentration in the blood is one of the indicators of the effect of the proposed transducer.

To reduce the risk of mechanical lung injury, efforts were made to shorten the exposure time of low-frequency ultrasound. In this present work, the effect of low-frequency ultrasound on the blood was investigated. Blood is a shear-thinning fluid with a complex reaction that is highly dependent on the ability of red blood cells (RBCs) to form aggregates. The RBC aggregates dissociate into single RBC cells when acoustic signal-induced shear forces exceed γ = 5–10 s^−1^ [21]. The effect of low-frequency ultrasound on biological cells, occurring at a lower temperature than high-frequency ultrasound, increases oxygen affinity and, consequently, a closer relationship with a higher oxygen saturation.

The presented graph (Fig. 8) proves the therapeutic effect of low-frequency ultrasound on the lungs, resulting in an increase in oxygen concentration in pulmonary blood. Oxygen is a pulmonary vasodilator. Although the treatment for PH focuses on pulmonary vasodilation with oxygen, long-term O_2_ therapy is not recommended unless patients develop hypoxemia, which is one of the leading causes of multiple organ injury and death in COVID-19 patients. The evidence presented in [22] suggests that O_2_ is a vasodilator in normoxic patients, and that it is not only related to blood-borne (oxyhemoglobin) mechanism, but also to alveoli, and that the therapeutic benefit of O_2_ is independent of the arterial oxygen level of 75 to 100 mm mercury (mmHg). This indicates that O_2_ is therapeutically beneficial for PH patients. The main cause of death from PH is right ventricular failure [23]. Chronic heart failure develops because of cardiac energy depletion due to oxygen deprivation at the mitochondrial level of cardiomyocytes. Almost 50% of patients with PH develop iron deficiency [24]. Iron deficiency, due to chronic blood loss or inadequate dietary iron absorption, causes iron deficiency anaemia, while inflammation-induced iron retention in innate immune cells and iron uptake blockade cause anaemia of chronic disease. Anaemia is defined as a decrease in the amount of RBCs, or hemoglobin, which carries oxygen in the blood. The amount of hemoglobin, which carries oxygen to the body's cells from the lungs, improves the absorption of iron [25]. Our finding [26] that low-frequency ultrasound dissociates aggregated erythrocytes into single erythrocytes, whose hemoglobin molecules interact with oxygen over the entire erythrocyte surface area than the aggregates of erythrocytes that are not exposed to ultrasound.

According to research in [27], reduced gas transmission in patients with PH has traditionally been associated with progressive pulmonary arterial attrition and vascular remodelling, which reduces the volume of capillary blood available for gas exchange. Ultrasonic exposure can also be used in clinical applications as focused therapeutic ultrasound with higher acoustic power, using contrast agents, known as microbubbles, for more effective drug absorption. Such improved absorption of drugs into cells is due to the increased permeability of the membrane under the influence of ultrasound [28]. The results of therapeutic drugs delivery using thoracic ultrasound and microbubbles, primarily to damaged areas of the lung, especially to the endothelium, have been described in a study [29]. Using our proposed low-frequency ultrasound transducer, it is possible to select an excitation frequency that coincides with the resonant frequency of the microbubbles, which results in their disruption, thereby accelerating drug delivery. This is also confirmed by the author of [30], who reported that low-frequency 20 kHz ultrasound in vitro (125 mW/cm^2^, 100 ms. pulses per second) increased the permeability of salicylic acid through human skin by almost 1000 times compared to high-frequency 1 MHz ultrasound (92 W/cm^2^). Acoustic cavitation, i.e., the formation and violent collapse of gas microbubbles in a liquid irradiated with low-frequency ultrasound, is a major contributor [31]. Cavitation in the lungs is undesirable because it can destroy the structure of the lung alveoli. In our research the level of cavitation was evaluated by the intensity of the acoustic signal causing the cavitation with mechanical index values greater than 0.6. From the equation to calculate mechanical index *MI* = *P*^*r.3*^*/√f* [5] where *P*^*r.3*^ stands for the rarefactional pressure (in unit of MPa) of the ultrasound field with an attenuation coefficient of 0.3 dB (MHz cm)^–1^ and *f* means the frequency (in unit of MHz) of the ultrasound wave. In the case of sheep, we used low-frequency and low-intensity ultrasound: max acoustic pressure measured was 75 kPa (see Table 2), frequency of sonication was 38 kHz and calculated *MI* = 0.075/0.195 = 0.385 < 0.6. The probability of cavitation was therefore very low.

High doses of oxygen cause pulmonary oedema and interstitial fibrosis, whereas continuous exposure to inhaled oxygen does not cause either epithelial damage or alveolar fibrosis [32]. Unlike breathing concentrated oxygen, which produces high levels of oxygen in the blood that can damage and kill cells, mainly in the eyes, the central nervous system and the lungs, the gradual change in blood oxygen levels caused by exposure to low-frequency ultrasound is similar to human exercise and is considered to be beneficial to the body. Pulmonary vasodilators contain nitric oxide (NO), which is essential for improving oxygen saturation, protecting cells, and preventing inflammation [33]. Inhaled NO is rapidly oxidized to NO_2_, which interacts with water to form highly harmful nitric acids that can cause pulmonary oedema and pneumonitis. The positive effects of inhaled NO may give short-term benefits, whereas rapid withdrawal of inhaled NO causes a rebound phenomenon. This is probably due to endogenous NO inhibition, leading to reduced oxygenation and increased pulmonary arterial pressure, so slow and gradual withdrawal of inhaled NO is recommended. Ultrasound-induced release of nitric oxide from tissues in patients with PH may be beneficial for oxygenation. Pulmonary vascular remodelling is a major structural change in the vascular wall that is dangerous in pulmonary hypertension. The endothelium is one of the inner layers of the blood vessel wall, lined with endothelial cells. The endothelium is an endocrine organ, that plays a major role in the regulation of angiogenesis, immune responses, and inflammation. A study [34] showed that low-frequency ultrasound increased the expression of vascular endothelial growth factors, and influenced the process of neovascularization in a diabetic rat model by promoting the differentiation and proliferation of endothelial fibroblasts. Low-frequency ultrasound has been shown to influence arterial and venous dilation and promote tissue perfusion [35]. In vivo studies in humans have shown that arterial dilation can be observed 1 min. after exposure to ultrasound, partly due to ultrasound-induced release of NO from the tissues, suggesting that ultrasound-mediated vasodilatation in PH patients results in an increase in gas transport.

Sudden cardiac death is the main genesis caused by PH. Dysfunction of the cardiac autonomic system is assessed by heart rate variability. A low heart rate is favourable in healthy individuals. The changes in pulse rate induced by low-frequency ultrasound are illustrated in Fig. 9. Heart rate recovery after exercise is an important indicator of the condition of patients with heart and lung diseases. The study in [36] presents the data on heart rate recovery in PH patients after a 6-min walk test. The research shows that heart rate recovery is an easily measurable clinical biomarker in the near-term disease space, which allows prediction of worsening clinical survival and hospitalization in patients with PH. The results of our study, which show a marked reduction in pulse rate after 7 min of ultrasound exposure, may provide an alternative for hospitalized patients who cannot walk.

Only a small increase in pulmonary pressure is one of the risk factors predicting the trajectory of clinical outcomes of patients. The presented graph (Fig. 10) supports the assumption that low-frequency ultrasound has a therapeutic effect on the lungs, leading to a reduction of pulmonary blood pressure. When exposed to low-frequency ultrasound, the erythrocytes in the aggregates dissociate into single erythrocytes [26] separated by a distance from each other and their number per unit volume of blood is reduced due to the gaps between them compared to the erythrocytes in the aggregates in blood not exposed to ultrasound. Therefore, when ultrasound exposure is discontinued, blood pressure recovers within a minute because the increase in the number of aggregated erythrocytes per unit volume, known as erythrocytosis, is accompanied by an increase in the viscosity of the blood and a recovery of blood pressure to the level it was before the ultrasound exposure. In PH, the heart pumps blood from the right ventricle to the lungs via blood vessels with increased muscle content in their walls. The small distance of the lungs from the right ventricle results in low blood pressure on this side of the heart and in the artery. The pressure is usually much lower than the systolic or diastolic blood pressure.

The research presented in [37] suggests that in pulmonary arterial hypertension, increased vascular pressure is associated with pulmonary artery vascular remodelling, small-artery obstruction, and increased vascular resistance to pulmonary blood flow. In the long term, high blood pressure can damage the heart and lead to right ventricular failure and, eventually, death.

A study in [38] shows that a 10 min treatment with low-frequency ultrasound can reduce the white-coat effect associated with anxiety about healthcare visits and improve clinical hypertension to acceptable blood pressure. The article [39] reported that low-frequency ultrasound induced a significant blood pressure decrease in hypertensive rats. After 1 week of total ultrasound exposure of 20 min/day, the systolic blood pressure of the rats decreased from 170 ± 4 mmHg to 128 ± 4.5 mmHg. Ultrasound stimulation did not cause significant tissue damage, cell apoptosis, or haemorrhage.

Vasodilator drug therapy is generally unable to selectively reduce pulmonary vascular resistance during long-term administration and frequently leads to systemic hypotension, exacerbation of pulmonary hypertensive state, worsening of right ventricular failure and systemic arterial desaturation. As a result, the average survival rate of pulmonary hypertension patients remains below 3 years after diagnosis. Extensive research efforts have led to the emergence of innovative treatments, such as stem cell therapy, gene transfer, and epigenetic therapy. The described innovative methods of pulmonary hypertension therapy, in contrast to our proposed application of low-frequency ultrasound, are invasive and expensive, requiring complex techniques and qualifications.

### Limitations and future works

Ultrasound can cause some biophysical effects, including thermal and non-thermal effects on cells, but these effects are significantly less in the case of low-frequency ultrasound than in the case of high-frequency. Sonoporation, the most widely studied non-thermal biological effect of ultrasound, is considered the basis of new therapeutic applications.

The clinical trials of this innovative low-frequency ultrasound transducer tested in sheep will continue with the goal of obtaining approval for the treatment of pulmonary hypertension in humans.

## Conclusion

The developed low-frequency ultrasound transducer is novel, as there is no evidence of transducers on the market that can excite higher vibrational modes, which would not only increase the penetration or acoustic pressure of the ultrasound acoustic signal, but also allow more precise targeting of the therapeutic effect. Measurements of the physiological parameters (blood oxygen concentration, pulse rate and blood pressure in the lungs) of anesthetized sheep were performed and show changes over 10% under the influence of low-frequency ultrasound after 7 min of sonication. The findings from the in vivo tests conducted on sheep demonstrate that the proposed low-frequency ultrasound transducer has the potential to noninvasively exert therapeutic effects on individuals with pulmonary hypertension. This approach can enhance their quality of life and potentially reduce their reliance on medication.

## Data Availability

Essential datasets supporting the conclusions are included in this published article.

## Abbreviations

PH: Pulmonary arterial hypertension
NO: Nitric oxide
O_2_: Oxygen
